# MAP: evaluation and multi-agent enhancement of large language models for inpatient pathways

**DOI:** 10.1038/s44401-026-00085-0

**Published:** 2026-06-01

**Authors:** Zhen Chen, Zhihao Peng, Xusheng Liang, Cheng Wang, Peigan Liang, Linsheng Zeng, Minjie Ju, Yixuan Yuan

**Affiliations:** 1 Department of Data Science and Artificial Intelligence, The Hong Kong Polytechnic University, Kowloon, Hong Kong SAR; 2 Department of Electronic Engineering, The Chinese University of Hong Kong, New Territories, Hong Kong SAR; 3 Guangzhou Hospital of Integrated Traditional Chinese and Western Medicine, Guangzhou, China; 4 Shenzhen Traditional Chinese Medicine Hospital, Shenzhen, China; 5 Department of Critical Care Medicine, Zhongshan Hospital Fudan University, Shanghai, China

**Keywords:** Health care, Diagnosis

## Abstract

Inpatient pathways require complex clinical decision-making based on comprehensive patient information, yet research on medical LLMs is limited in this area due to the lack of large-scale datasets. Existing medical benchmarks primarily focused on question-answering and examinations, overlooking the multifaceted nature of inpatient decision-making. To address this gap, we developed the IPDS benchmark, comprising 51,274 cases across 9 triage departments, 17 major disease categories, and 16 treatment options. We further proposed the Multi-Agent Inpatient Pathways (MAP) framework, containing three specialized clinical agents: a triage agent for patient admission, a diagnosis agent for diagnostic decision-making, and a treatment agent for care planning. A chief agent guides and promotes these agents to ensure coordination. Experiments demonstrated that MAP achieved superior alignment with operational protocols compared to state-of-the-art LLMs. The MAP sets a foundation for advancing inpatient support systems, offering significant potential for enhancing operational efficiency and resource planning in healthcare facilities.

## Introduction

As a critical component of worldwide healthcare systems, inpatient pathways^1–10^ are characterized by the necessity for clinical decision-making based on comprehensive patient information, and present challenges for clinicians across all levels of experience. Even experienced clinicians encounter challenges in the inpatient pathways, where the complexity of clinical decision-making in time-sensitive situations can affect diagnostic accuracy. It is suggested that diagnostic errors lead to 40,000 to 80,000 fatalities annually, affecting more than 250,000 Americans who encounter such errors while receiving care in hospitals in the United States^11^. The consequences of diagnostic errors can lead to a cascade of adverse outcomes, ranging from unnecessary treatments and prolonged hospital stays to disability or even death, highlighting the critical importance of reliable clinical decision support systems in healthcare^12–16^. With the breakthrough of large language models (LLMs), recent studies have demonstrated promising capabilities in medical knowledge retrieval^17^^,^^18^, consultation systems^19^^,^^20^, and diagnostic suggestions^21–23^. LLMs can leverage publicly available medical knowledge to enhance medical reasoning and comprehension, demonstrating the potential to rival clinicians in medical licensing examinations^24^. Additionally, LLMs can translate complex medical terminology into plain words, making physician-patient interactions more efficient by clearly explaining medical procedures and treatment options^25^. However, the effectiveness of LLMs in supporting inpatient pathways still remains unexplored. Critically, effective inpatient management extends beyond pathological diagnosis, and it requires precise operational decision-making to optimize patient flow and resource allocation, e.g., to determine which department is suitable to handle the specific patient. Especially, the complex inpatient pathways demand sophisticated capabilities, including the systematic analysis of diverse and complex clinical information from electronic health records, prioritization of critical issues within the dynamic and evolving nature of inpatient settings, and support for nuanced diagnostic decision-making within these complex clinical environments.

**Fig. 1:**
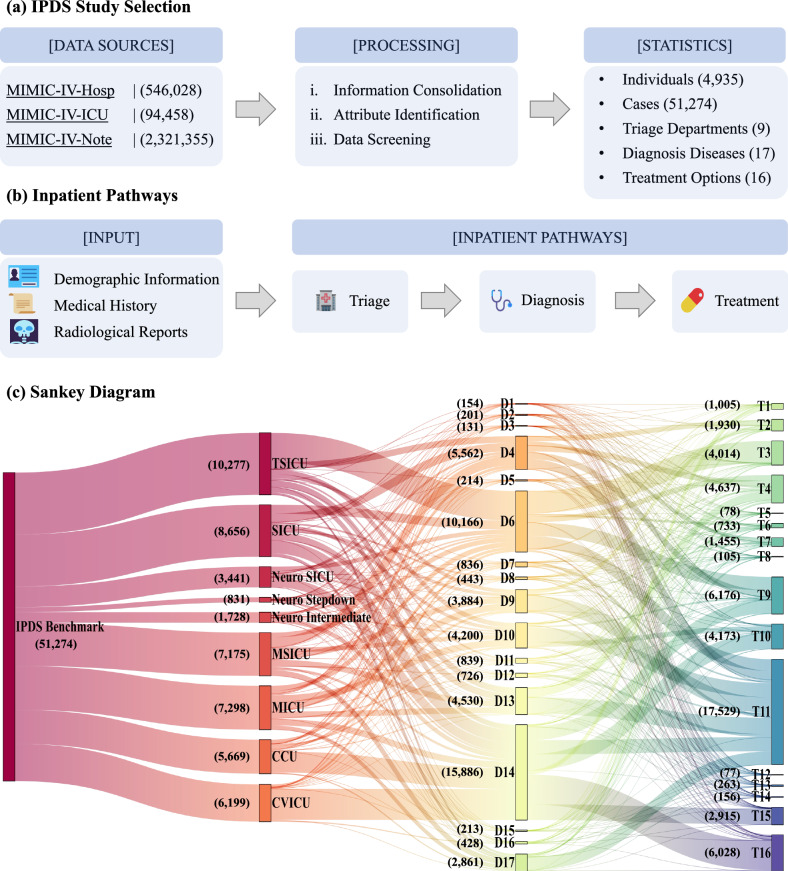
Illustration of the inpatient pathway decision support (IPDS) benchmark. **a** The statistics, processing, and data sources of the IPDS benchmark. The IPDS contains 51,274 cases across 9 departments, 17 diseases (D1–D17), and 16 treatments (T1–T16), and provides a comprehensive evaluation of LLMs in different inpatient scenarios. **b** The evaluation of the IPDS benchmark. **c** The Sankey diagram of the IPDS benchmark. This diagram visualizes the data distribution in the workflow of different inpatient scenarios. The specific abbreviations and details of the department, disease, and treatment options are provided in detail.

To fill these gaps, we developed the Inpatient Pathway Decision Support (IPDS) benchmark by systematically integrating comprehensive clinical data of the Medical Information Mart for Intensive Care (MIMIC)-IV database at the Beth Israel Deaconess Medical Center^26–34^. Unlike existing medical benchmarks^16^^,^^19^^,^^35–39^ that primarily focused on medical licensing exams and general clinical questions, the IPDS benchmark encompasses 51,274 patient cases across 9 clinical departments, 17 disease categories, and 16 standardized treatment options. We clarify that the primary goal of this study is to assist in operational workflows rather than replacing clinician-level pathological diagnosis. In this context, the labels serve as coarse-grained operational categories derived from standardized hospital records. Our data pipeline utilized an international disease statistical classification list^40^ to recategorize 1298 original disease labels into 17 broader categories. As such, the IPDS incorporates curated demographic information, radiological reports, and medical history, which are essential for comprehensive inpatient pathway decision support, providing a comprehensive representation of inpatient scenarios, as shown in Fig. 1. We empirically observed that state-of-the-art LLMs merely achieved unsatisfying performance on inpatient scenarios of the IPDS, e.g., the general LLaMA3-8B with 49.30%, InternLM2-20B with 51.70%, and the medical HuatuoGPT2-13B with 53.00% in the accuracy of the diagnosis task. These findings indicate that the state-of-the-art LLMs can hardly meet the requirements of inpatient scenarios, resulting in an urgent need for a diagnosis support framework with significantly improved inpatient pathway performance.

**Fig. 2:**
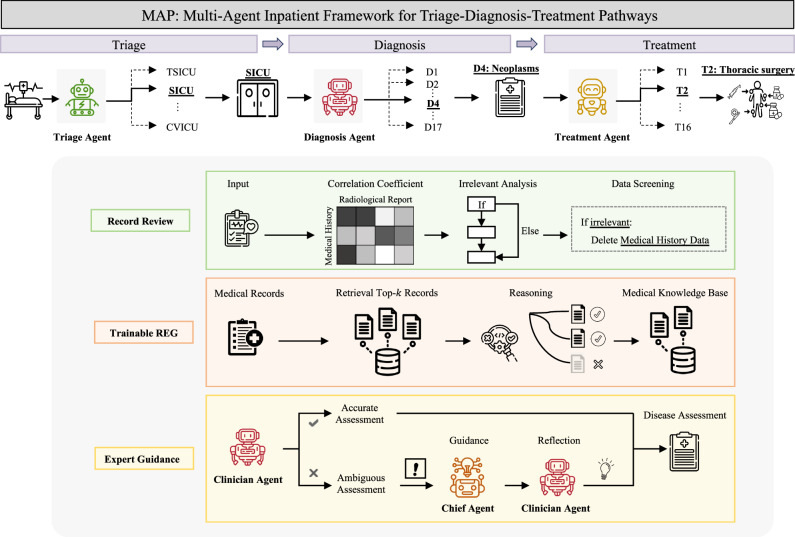
Overview of the Multi-Agent Inpatient Pathways (MAP) framework. The MAP is a multi-agent collaborative framework that simulates the inpatient pathway flow. The framework consists of LLM-empowered agents: a triage agent for department triage, a diagnosis agent for each department for the clinical decision-maker, a treatment agent for the treatment plan, and a chief agent for overseeing diagnosis and treatment pathways. Three key components support our MAP framework: (1) a record review module that analyzes patient data, including demographic information, radiological reports, and medical history; (2) a trainable REG module that integrates clinical knowledge bases with chain-of-thought reasoning to support reliable diagnostic decision-making; and (3) an expert guidance module that ensures diagnostic rigor through structured supervision of the diagnosis agent.

We then developed the Multi-Agent Inpatient Pathways (MAP) framework to support the Triage-Diagnosis-Treatment (TDT) clinical pathway, as illustrated in Fig. 2. Specifically, our MAP framework established the collaboration among three clinician agents and a chief agent, including the triage agent that manages the patient admission, the diagnosis agent that acts as the primary decision-maker in the department, the treatment agent that provides treatment plans, and the chief agent that oversees different clinical tasks along inpatient pathways. To promote collaboration among these agents, our MAP was elaborately developed with three modules. The record review module employed a semantic analysis component to comprehend medical terminology and clinical descriptions of patient data. The trainable retrieval-enhanced generation (REG) module retrieves the most relevant medical records from an extensive knowledge base, simulating the case review process of the diagnosis agent to maintain diagnostic accuracy. The expert guidance module achieves the supervisory relationship between the diagnosis agent and the supervisor chief agent of each department. The effectiveness of these modules was verified by the ablation studies in the experiments.

We conducted extensive studies on supporting inpatient pathways by comparing our MAP and state-of-the-art LLMs^41–45^ across three distinct inpatient scenarios of TDT. First, the *triage* task determines the most suitable department for patients based on symptoms, medical history, and urgency, involving high-level categorization into broad clinical areas. After that, the *diagnosis* task focuses on utilizing diagnostic information (e.g., demographic information, radiological reports, and medical history) to identify specific diseases or conditions, requiring in-depth analysis of clinical data. Finally, the *treatment* task aims to select proper treatment options considering the patient’s specific needs and condition severity, focusing on optimizing outcomes and minimizing risks, involving decision-making about interventions and follow-up care. Our findings indicate that the proposed MAP framework significantly enhances the capabilities of LLMs in supporting inpatient pathways, particularly in complex disease cases where state-of-the-art LLMs exhibited unsatisfactory performance. Furthermore, we examined our MAP and state-of-the-art LLMs concerning diagnostic challenges across various disease categories, analyzed the conditions leading to misdiagnosis, and evaluated diagnostic performance while excluding specific inputs. We further randomly sampled 100 cases from the IPDS test set and conducted the statistical analysis of clinicians’ diagnostic performance, assessing the alignment with operational protocols of our MAP and advanced LLMs with expert judgments. To rigorously validate the system’s generalizability beyond the MIMIC-IV database, we additionally constructed and evaluated a private external cohort comprising 150 inpatient records from an international hospital, where our MAP framework demonstrated robust transferability of clinical logic. Notably, the MAP framework is designed to be fundamentally label-agnostic, which ensures that the pipeline can be adapted to other clinical taxonomies. In summary, our MAP framework, benefiting from a diagnostic architecture specifically designed for the inpatient pipeline, improves the AI system to provide more accurate and effective inpatient support. We have made our IPDS and MAP publicly available to facilitate further research in inpatient diagnostic support.

## Results

### The IPDS benchmark

To address the critical need for evaluating LLMs in supporting inpatient pathways, we introduced the IPDS, a novel benchmark systematically integrated from the MIMIC-IV database, specifically from MIMIC-IV-Hosp, MIMIC-IV-ICU, and MIMIC-IV-Note databases of the Beth Israel Deaconess Medical Center^26^^,^^31^^,^^32^. The IPDS encompasses 51,274 patient cases across 9 departments, 17 major disease categories, and 16 standardized treatment pathway options, as shown in Fig. 1. We compared our IPDS with existing clinical benchmarks in Table 1, in terms of the source type, number of samples, and multiple-department involvement. Unlike state-of-the-art medical benchmarks^46–49^, the IPDS is elaborately designed for inpatient diagnostic support, incorporates comprehensive patient data (e.g., demographic information, radiological reports, and medical history) to complete three distinct clinical classification tasks, including triage, diagnosis, and treatment pathways. Specifically, the triage task is simplified as a nine-category classification task aiming to correctly evaluate the triage agent’s ability to direct patients to the appropriate department. The diagnosis task is a multi-class classification task with seventeen disease categories, which is a critical bridge between the initial department assignment and the subsequent treatment pathway in inpatient care. The treatment task is a multi-category classification task with sixteen treatment options designed to identify whether the patient is receiving the appropriate treatment. To this end, the multifaceted structure of the IPDS benchmark allows for a thorough assessment of LLM capabilities in three critical areas, including medical information analysis, consolidation, and diagnostic decision-making, which is essential to inpatient diagnostic support. The development of the IPDS benchmark followed rigorous data sanitization protocols^50^^,^^51^ and access guidelines^52^^,^^53^ to ensure patient privacy protection and data integrity. These measures made the IPDS a valuable and responsible resource for advancing LLMs in inpatient diagnostic support. We split 1000 samples of IPDS to evaluate the model’s ability to support inpatient pathways for triage, diagnosis, and treatment tasks, and the remaining samples were used for model training. Beyond this evaluation, the IPDS aims to facilitate the development of LLMs specifically designed for supporting inpatient pathways, establishing new standards for creating and assessing clinical decision support systems.

**Table 1:** Statistics comparison of existing benchmarks and our IPDS benchmark, including the source type, number of samples, and multiple departments' involvement

Benchmark	Data source	Samples	Multiple departments
PubMedQA^84^ [EMNLP’19]	Public	500	✗
MedQA^35^ [ASci.’21]	Public	1273	✗
CMB-Clin^85^ [NAACL’24]	Public	74	✗
HealthSearchQA^86^ [Nature’23]	Public	3173	✗
CMExam^87^ [NeurIPS’24]	Public	68,119	*✓*
MedBench^38^ [AAAI’24]	Private	1025	✗
ClinicalBench^88^ [ArXiv’24]	Private	1500	*✓*
MIMIC-CDM^5^ [Nature Medicine’24]	Public	2400	✗
IPDS (Ours)	Public	51,274	*✓*

In general, the main shortcomings of existing evaluation benchmarks include (1) lack of comprehensive and evenly distributed departmental coverage to prevent evaluation bias; (2) data sources often come from easily accessible online consultation platforms, medical textbooks, and professional examinations, which poses high risks of data leakage; (3) existing benchmarks primarily test medical knowledge through multiple-choice questions, which differ significantly from real-world diagnostic scenarios.

### The experimental settings

To assess the capability of varying LLMs in supporting inpatient pathways, we evaluated the general LLMs (InternLM2-7B/20B^44^, LLaMA3-8B/70B^45^), the specialized medical LLMs (Clinical-Camel-70B^43^, Meditron-70B^42^, LLaMA3-Med-8B^54^, HuatuoGPT2-7B/13B^41^, HuatuoGPT-o1-8B^55^), and our MAP framework. Due to privacy concerns and data agreements (https://physionet.org/about/licenses/physionet-credentialed-health-data-license-150/https://physionet.org/licenses), the MIMIC-IV database prohibits the use of its data with external APIs such as OpenAI or Google, preventing the IPDS research from evaluating ChatGPT^56^, GPT-4^57^, and Med-PaLM^58^. Our systematic studies reveal several significant findings regarding the capabilities and limitations of LLMs in supporting inpatient pathways.

### The underperformance of state-of-the-art LLMs in supporting inpatient pathways

**Fig. 3:**
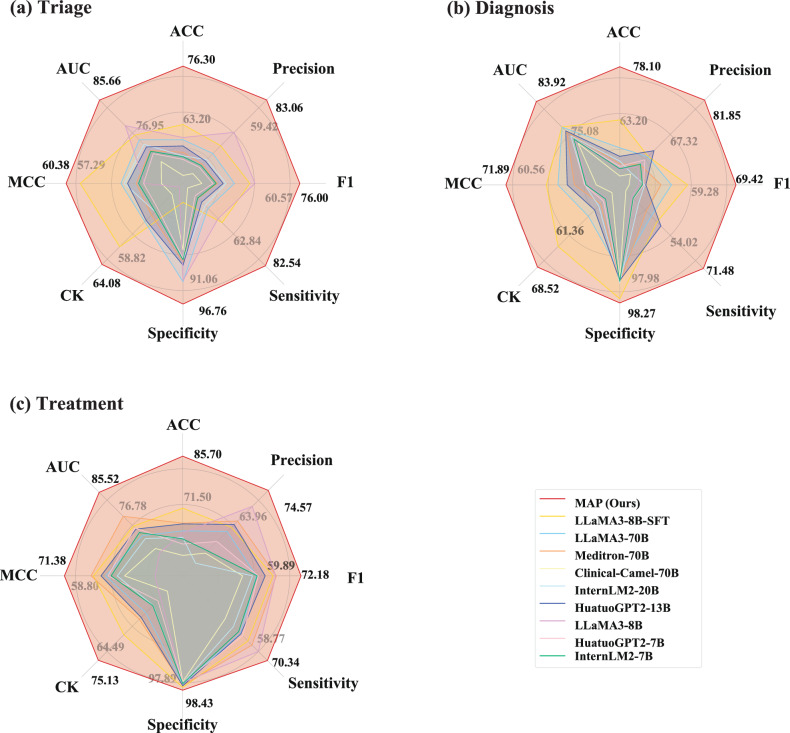
The MAP demonstrated enhanced capabilities in supporting inpatient pathways compared to state-of-the-art LLMs. **a** Triage, **b** diagnosis, and **c** treatment. Current general LLMs showed limited performance in diagnostic support, such as LLaMA3-70B with 68.08% accuracy, while specialized medical models, such as Clinical-Camel-70B, with 47.50% accuracy. The MAP achieved an overall diagnostic support accuracy of 78.10%, showing improvements of 30.60, 27.20, and 25.10% over Clinical-Camel-70B, Meditron-70B, and HuatuoGPT2-13B (*p* < 0.001 for all comparisons). The best and second-best performance values are marked for better clarity. These results demonstrated the potential of the MAP as a clinical decision-support tool across diverse inpatient scenarios.

Our analysis revealed significant limitations in the ability of state-of-the-art LLMs to support inpatient pathways across triage, diagnosis, and treatment tasks. As illustrated in Fig. 3, we compared the classification performance of our MAP and state-of-the-art LLMs on these three common clinical classification tasks, where we can find that state-of-the-art LLMs have demonstrated unsatisfying performance in inpatient scenarios within the IPDS. For example, LLaMA3-8B achieved only 60.30%, 49.30%, and 64.80% accuracy in triage, diagnosis, and treatment inpatient pathway tasks, respectively. Similar limitations exist in specialized medical LLMs with HuatuoGPT2-13B (58.40%, 53.00%, 67.20%), Clinical-Camel-70B (51.80%, 47.50%, 58.60%), and Meditron (57.00%, 50.90%, 67.40%). Even the LLaMA3-8B-SFT, i.e., LLaMA3-8B fine-tuned by the instruction tuning on the IPDS benchmark, has only inferior performance (63.20%, 63.00%, 71.50%). These results revealed the substantial potential for improvement in supporting the inpatient diagnostic capabilities of LLMs.

**Fig. 4:**
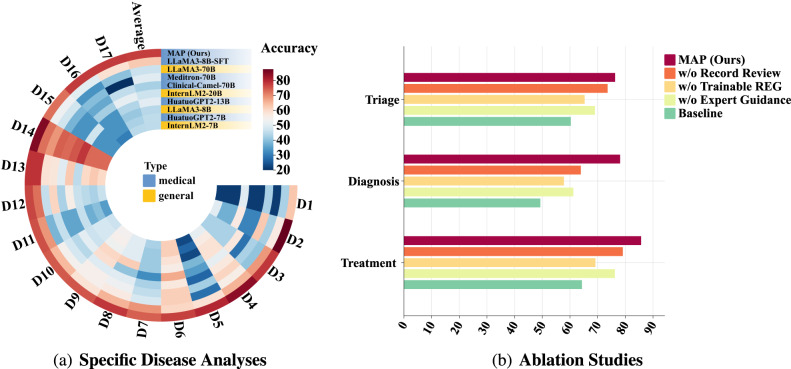
State-of-the-art LLMs demonstrated unsatisfying inpatient diagnostic support capabilities for complex inpatient clinical cases; in contrast, MAP enhances that capability. **a** For instance, HuatuoGPT2-13B performed a low accuracy of 38.46% in D5 (mental and behavioral disorders). The MAP showed significant improvement in supporting the inpatient pathways, achieving an accuracy of 79.86% in D5. **b** Such an enhancement was attributed to the integration of our proposed record review, trainable REG, and expert guidance modules, verified by the corresponding ablation studies.

As shown in Fig. 4a, state-of-the-art LLMs demonstrated notable limitations in supporting the diagnosis of complex clinical presentations involving multiple organ systems. Particularly challenging areas include D5 (mental and behavioral disorders) and D9 (diseases of the respiratory system), where the accuracy falls below 42.31%. These findings highlighted the need for specialized diagnostic support systems designed specifically for the inpatient setting, which motivated the development of our MAP framework.

### The MAP enhances the capability of inpatient diagnostic support, particularly in complex disease cases

We developed the MAP, a novel multi-agent collaboration framework specifically designed to support inpatient pathways, as illustrated in Fig. 2. The MAP framework demonstrated significant improvements across all evaluation metrics, as shown in Fig. 3. In particular, MAP achieved an overall diagnosis accuracy of 78.10%, reflecting a 28.80% improvement over LLaMA3-8B, which had an accuracy of 49.30%. Notably, the MAP framework outperformed the best specialized LLM, HuatuoGPT-o1-8B, by a 22.30% improvement in accuracy (i.e., 78.10% vs. 55.80%).

The MAP framework exhibited significant performance in diagnosing complex conditions. For instance, as shown in Fig. 4a, for D5 (mental and behavioral disorders), the MAP achieved an accuracy of 79.86%, representing a 21.22% improvement over the second-best model, LLaMA3-8B-SFT, which scored 58.64%. This performance improvement can be attributed primarily to the design of the record review module, trainable REG module, and expert guidance module, whose effectiveness is verified in Fig. 4b. For readability, more ablation results toward more evaluation metrics were given in the Fig. 9. Benefiting from the reasoning process and diagnostic results recorded by the trainable REG module, MAP also offers explainable and clinically relevant diagnostic support.

### Clinical validation of MAP with significant protocol alignment

Additionally, we conducted a statistical analysis of the diagnostic performance and decision-making abilities of clinicians by randomly selecting 100 cases from the IPDS test set to create a test set in a multiple-choice question format. Specifically, three board-certified clinicians from different tertiary hospitals were recruited to independently evaluate the patient cases, providing their reasoning and diagnostic results, where Clinician-15yr, Clinician-10yr, and Clinician-5yr represent clinicians with 15, 10, and 5 years of clinical experience, respectively. To guarantee a fair and rigorous comparison, these clinicians independently evaluated 100 randomly sampled cases. Crucially, they were provided with the exact same clinical notes that were used as inputs for the LLMs, ensuring a strict peer evaluation. Utilizing their extensive clinical experience, the clinicians were required to provide the three most likely diagnoses and rank them according to their likelihood, facilitating the subsequent analysis.

**Fig. 5:**
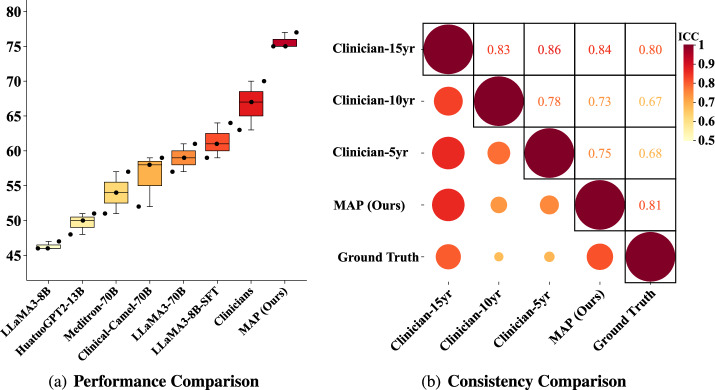
Performance and consistency comparison of LLMs, clinicians, and MAP in supporting patient pathways through the IPDS benchmark. **a** Performance comparison: the accuracy of different LLMs, clinicians, and the proposed MAP is shown. MAP (ours) demonstrates the highest performance with minimal variance, achieving 9% higher accuracy than board-certified clinicians. **b** Consistency comparison: MAP achieves strong agreement with the ground truth (ICC = 0.81), exceeding the agreement levels of individual clinicians with the ground truth (ICC ∈ [0.67, 0.68]). MAP also aligns strongly with clinicians (ICC ∈ [0.75, 0.84]). The size and color of the circles represent the ICC values, with darker colors and larger circles indicating higher consistency.

As shown in Fig. 5a, the MAP demonstrated superior performance compared to both the general and medical LLMs as well as three board-certified clinicians, achieving the highest accuracy in diagnostic tasks. In contrast, the general LLM LLaMA3-8B and the medical LLM HuatuoGPT2-13B demonstrated significantly lower performance, with accuracy levels 27% to 30% below those of MAP. Notably, the temperature parameter of the LLM introduces randomness into the model’s classification performance. Furthermore, as illustrated in Fig. 5b, the inter-rater reliability analysis revealed the degree of diagnostic correlation among three clinicians, MAP, and the ground truth. The MAP achieved a strong agreement with the ground truth, as indicated by an intra-class correlation coefficient (ICC) of 0.81, which exceeded the agreement levels between individual clinicians and the ground truth (ICC ∈ [0.80, 0.67, 0.68]). Additionally, the MAP maintained a strong alignment with clinician assessments (ICC ∈ [0.84, 0.73, 0.75]), especially rivaling the diagnosis results of experienced clinicians with 15-year clinical experience, maintaining significant alignment with operational protocols.

### Generalization on international inpatient records

To assess the generalization of the proposed MAP across diverse healthcare settings, we constructed a high-quality validation cohort comprising 150 inpatient records randomly selected from Zhongshan Hospital, Fudan University, spanning 2022 to 2024. To ensure the rigorousness of this validation, the standardized labels were manually annotated by two senior clinicians from the respective hospital, where these experts strictly followed the same label codebook and inclusion criteria used for the IPDS benchmark, explicitly mapping confirmed discharge diagnoses to our standardized taxonomy. Importantly, the MAP was trained solely on the training set of the IPDS benchmark, derived from MIMIC-IV data^31^, and evaluated via zero-shot inference with frozen parameters. To bridge the linguistic gap^59^ and isolate the clinical reasoning capability, the raw Chinese EHR notes underwent a rigorous translation-and-verification pipeline: initial translation was performed using Google Translate^60^, and the translated texts were reviewed by bilingual clinical experts to ensure clinical terminology was accurately preserved. This preprocessing step aligns the input feature space with the MIMIC-IV training data, ensuring the evaluation tests the robustness of clinical logic and workflow pattern transfer.

During this validation process, we observed several concrete structural and stylistic differences between the Chinese EHR dataset and MIMIC-IV that extend beyond mere linguistic translation. First, regarding documentation style and temporal granularity, the Chinese EHR data frequently preserves precise long-term chronological details, such as recording *40 years of hepatitis B*, and actual ages up to 102 years. In contrast, MIMIC-IV focuses predominantly on the current acute admission and heavily de-identifies ages over 89. Second, we noted differences in how medical interventions are documented. In the Chinese EHR, prior interventions are frequently embedded directly into the diagnosis string (e.g., *post-ERBD* or *post-stent implantation*), whereas MIMIC-IV typically decouples these into separate procedure tables, such as procedures_icd. Third, divergences in systemic classification logic were evident. For instance, while *severe pneumonia* maps to D9 (diseases of the respiratory system), in our English-based taxonomy, clinicians in the Chinese dataset often document and treat it under the clinical logic of D17 (certain infectious and parasitic diseases).

Nevertheless, we emphasize that while our translation-and-verification pipeline, guided by bilingual clinical experts, successfully minimized semantic loss, these underlying differences in clinical documentation logic remain a key factor in interpreting cross-system performance. Despite these granular structural discrepancies and different comprehensive diagnosis styles, the high performance achieved by MAP on this international cohort primarily validates the robust transferability of the underlying clinical logic and workflow patterns across healthcare systems, rather than demonstrating perfect linguistic mapping or complete invariance to documentation practices. For comparison, we included several state-of-the-art general-purpose and medical-domain LLMs. As illustrated in Table 2, the comparison demonstrates that the MAP framework achieved an accuracy of 80.67%, outperforming all baseline models, including HuatuoGPT-o1-8B (72.00%) and LLaMA3-8B-SFT (75.33%). These findings underscore the robustness of the multi-agent mechanism, enabling it to maintain high diagnostic accuracy even in datasets from entirely different medical systems once the language barrier is removed. This consistent performance across international clinical data validates the applicability of MAP as a reliable framework for global inpatient pathway decision support.

**Table 2:** Comparison of the diagnosis task on the private inpatient dataset

Method	Acc	F1	AUC	PRE	SEN	SPE	CK	MCC
LLaMA3-8B^45^	52.67	51.42	56.08	53.92	50.83	54.50	5.08	5.25
LLaMA3-Med-8B^54^	65.33	64.25	69.41	66.72	63.83	66.83	29.87	30.01
Clinical-Camel-70B^43^	54.00	52.74	57.33	55.22	52.17	55.83	7.83	8.00
Meditron-70B^42^	58.00	56.88	61.75	59.47	56.33	59.67	14.92	15.08
LLaMA3-70B^45^	68.00	67.22	72.08	69.85	66.83	69.17	35.17	36.42
InternLM2-20B^44^	63.33	62.14	66.82	63.88	61.74	65.17	26.12	26.18
HuatuoGPT2-13B^41^	68.67	67.91	72.79	70.52	67.50	69.83	36.25	36.42
HuatuoGPT-o1-8B^55^	72.00	71.06	75.93	73.52	70.33	73.67	43.12	43.25
LLaMA3-8B-SFT^45^	75.33	74.81	79.12	76.94	74.50	76.17	49.83	50.12
MAP (Ours)	**80.67**	**80.25**	**84.92**	**82.33**	**79.83**	**81.50**	**60.42**	**60.67**

Best in bold.

## Discussion

Our systematic evaluation of the MAP framework in supporting inpatient pathways provided important insights into the potential and limitations of LLM-assisted inpatient pathways. We further investigated the patterns of MAP in diagnostic challenges across different disease categories, the relationship between clinical data quality and system performance, and areas for future development.

**Fig. 6:**
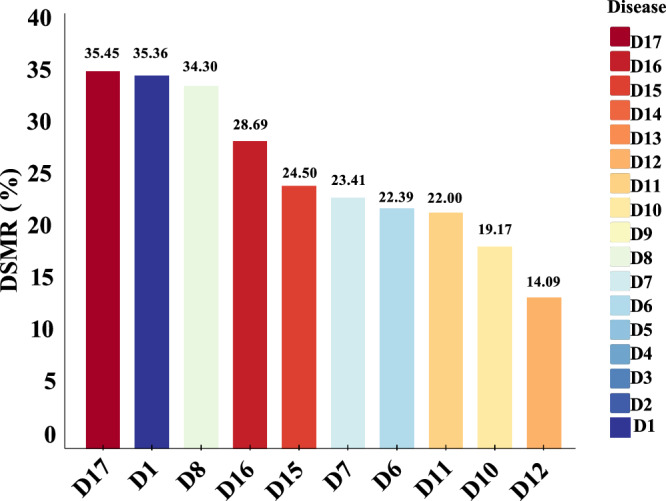
Different diagnostic challenges across disease categories in inpatient settings. We conducted the misdiagnosis rates comparison across the top 10 disease categories, where misdiagnosis rates ranged from 35.45% to 14.09%, demonstrating disease-specific diagnostic complexity. In particular, D1 (symptoms, signs, and abnormal clinical and laboratory findings, not elsewhere classified), D17 (certain infectious and parasitic diseases), and D8 (diseases of the skin and subcutaneous tissue) showed the highest rates (above 35.45%), highlighting the need for improved accuracy in these areas.

Through systematic analysis of disease misdiagnosis rates in Fig. 6, the study revealed significant differences in the difficulty of diagnosis across disease types. Among them, D17 (certain infectious and parasitic diseases) disease showed the highest misdiagnosis rate (35.45%), followed by D1 (symptoms, signs, and abnormal clinical and laboratory findings, not elsewhere classified) (35.36%), and D8 (diseases of the skin and subcutaneous tissue) (34.30%). This prominent stratification characteristic reflects that partial diseases face unique challenges in clinical diagnosis. Of particular note is that the top three high misdiagnosis rate diseases exceeded the 34.00% threshold, a finding highlighting the urgent need to enhance diagnostic accuracy in these specific disease areas. At the same time, the span from the highest misdiagnosis rate of 35.45% to the lowest misdiagnosis rate of 14.09% showed that the diagnosis complexity had apparent disease specificity.

**Fig. 7:**
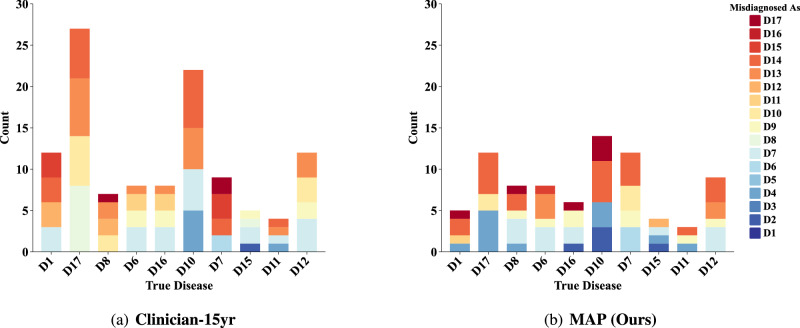
The MAP demonstrated superior diagnostic accuracy compared to the best-performing clinician. **a** Clinician-15yr; **b** MAP. In particular, the MAP significantly reduced false positives and exhibited a balanced misdiagnosis distribution across disease categories, and in contrast, the clinicians' errors were concentrated in specific categories. The colors in the bars represent the diseases that were misdiagnosed into different categories (D1 -D17).

We visualized misdiagnosis patterns between Clinician-15yr and the MAP toward evaluating different disease categories (D1–D17), where Fig. 7 shows the distribution of misdiagnosis for each disease category. We found that the MAP has superior diagnostic accuracy to Clinician-15yr, especially in reducing false positive samples and maintaining diagnostic specificity across different disease categories. Moreover, the MAP demonstrated consistently superior diagnosis across all disease categories. In particular, the MAP showed the most balanced misdiagnosis distribution, with consistently high diagnosis counts across all disease categories. The best-performance Clinician-15yr misdiagnoses were more pronounced in specific disease categories D1 (symptoms, signs, and abnormal clinical and laboratory findings, not elsewhere classified) and D17 (certain infectious and parasitic diseases) but with lower overall misdiagnosis counts, showing moderate performance with concentrated but significant misdiagnosis patterns. Crucially, these results suggest that the MAP framework offers distinct advantages in standardized healthcare settings. We explicitly acknowledge that achieving high accuracy on a specific label taxonomy does not equate to superior clinical reasoning compared to human doctors. However, while human decision-making inherently involves subjective variation, the MAP’s ability to *fit* the taxonomy reflects a high degree of operational consistency and adherence to the fixed coding ontology. We believe that this capability holds significant health systems value for tasks such as automated triage, billing code assignment, and resource planning, where reducing variability and ensuring standardization are the primary operational objectives.

**Fig. 8:**
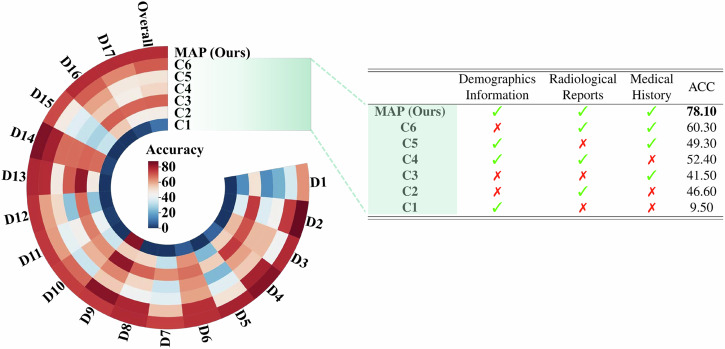
Impact of clinical data input on inpatient diagnostic support performance. (1) Comparison of comprehensive medical history improves diagnostic support accuracy from 23.08% to 79.86% for D5 (mental and behavioral disorders); (2) Incorporation of radiological findings enhanced diagnostic support accuracy by 31.03% (from 47.50% to 78.53%) for D13 (diseases of the digestive system); (3) Demographic information contributed a 5.80% improvement in overall diagnostic support accuracy (from 46.60% to 52.40%). These findings highlighted the importance of systematic integration of clinical data components, particularly emphasizing the value of medical history and radiological data in supporting inpatient diagnostic decisions.

We found that including or excluding specific input data could substantially affect the performance of LLMs, evidenced by ablation experiments of input components such as demographic information, radiological reports, and medical history on the IPDS benchmark toward inpatient pathways, as shown in Fig. 8. We observed that: (1) These input components played a critical role in integrating patients’ medical history for accurate diagnosis, particularly in complex, chronic conditions. The significant 55.02% increase in the MAP’s diagnostic accuracy, from 23.08% to 78.10%, for patients with D5 (mental and behavioral disorders), when medical history was included, is a reassuring validation of the value of our work since historical data provided essential context for interpreting current symptoms. (2) Radiological reports also play a pivotal role in enhancing diagnostic accuracy, especially for internal conditions. The 31.03% improvement from 47.50% to 78.53% in the diagnosis of D13 (diseases of the digestive system) when radiological report data were included. This underscores the importance of integrating imaging data into LLM-assisted inpatient pathways. (3) Our research found that while demographic information contributed to the diagnosis task, its impact was less pronounced than medical history and radiological report data. Including demographic information improved overall accuracy by only 5.80% (from 46.60% to 52.40%), suggesting that while relevant, this information plays a supporting role in inpatient pathways. These findings underscore the urgent need for comprehensive and diverse data inputs in LLM-assisted inpatient pathways. They also prioritize certain data types in time-critical situations, particularly medical history, and radiological reports, emphasizing the importance of these data types in enhancing diagnostic accuracy and the critical role of our research in addressing this need.

Our findings underscored the significant potential of our MAP framework in enhancing LLMs for inpatient pathways, showcasing the transformative possibilities of AI in healthcare. Despite these impressive results, our study also identified critical areas for improvement. First, the development of the MAP framework relies exclusively on the IPDS benchmark derived from MIMIC-IV records. Future iterations should incorporate larger-scale and more diverse datasets to optimize the framework, thereby strengthening the clinical decision-making capabilities of LLMs in both inpatient and outpatient diagnoses and further advancing the field of emergency medicine. Second, current models are limited to handling simple inpatient tasks. Increasing task complexity, such as integrating prognosis prediction and report generation, can assist medical professionals in reasoning through complex and less obvious cases, enhancing the interpretability of MAP decisions and driving progress in healthcare. Furthermore, existing models primarily address static tasks, whereas most clinical scenarios are inherently dynamic. Developing human-AI collaborative models will enable more efficient clinical judgment and remain essential for delivering optimal care in inpatient settings. In summary, our research provides a solid foundation for the future development of LLMs, with the ultimate aim of improving real-world patient outcomes in diverse and dynamic clinical environments.

Nevertheless, our international generalization validation highlights that beyond mere language barriers, fundamental structural and systemic differences between healthcare datasets inherently influence cross-language transferability and the interpretation of results. As observed in our validation cohort, variations in temporal density of U.S. databases^32^^,^^34^ can affect a model’s ability to capture long-term disease progression. Furthermore, the structural discrepancy of embedding interventions within diagnosis records requires the model to possess a higher level of semantic flexibility to recognize that a diagnosis in one system may be formally recorded as a procedure in another. This type of structural divergence is a persistent challenge frequently highlighted in cross-institutional EHR interoperability studies^61^. Finally, nuances in systemic classification logic present additional hurdles. The fact that diagnostic grouping logic differs across healthcare systems might introduce subtle biases when a model maps diverse, local clinical practices to a rigid, Western-centric classification hierarchy. This emphasizes that systemic differences in clinical coding and institutional protocols often pose significantly greater generalization barriers than the language itself^59^.

## Methods

### Benchmark preparation

We curated the IPDS, an inpatient diagnostic support dataset Table 3 integrating three information modules from the Medical Information Mart for Intensive Care (MIMIC)-IV database, namely MIMIC-IV-Hosp, MIMIC-IV-ICU, and MIMIC-IV-Note. Specifically, the MIMIC-IV-Hosp module, containing data on 546,028 unique hospitalizations for 223,452 individuals, provided demographic information (language, marital status, race, gender), diagnosis information (ICD code, ICD version, long title, disease)^62^, and treatment pathway options (current service). The MIMIC-IV-ICU module, with data on 94,458 ICU stays for 65,366 unique individuals, contributed hospital admission information, specifically the department distribution. The MIMIC-IV-note module included 331,794 discharge summaries and 2,321,355 radiological reports, where we extracted the medical history and radiological reports of patients.

To ensure data integrity and eliminate biases, we selected only the first hospital visit for each patient to construct the IPDS benchmark. This guarantees that the samples are independent at the patient level and avoids overlap or redundancy from subsequent visits. For each selected visit, only the information available up to the time of the first visit was included. This design ensures that the dataset focuses on real-world, forward-looking clinical decision-making, emulating the information available to clinicians at the time of a patient’s initial presentation. We began with data consolidation by identifying and handling missing values, such as filtering out samples with null radiological reports. Next, we conducted feature selection to identify the most relevant diagnostic features, including demographic information, radiological reports, and medical history. Finally, we performed comprehensive data screening to delete irrelevant distractors, such as medical history data that was unrelated to the radiological reports. To ensure methodological rigor and reproducibility, we formalized the label definitions of the triage, diagnosis, and treatment tasks, as elaborated in Tables 4–6, respectively. Specifically, the mapping relationship between diagnosis options and ICD codes is detailed in Table S1 of the Supplementary Material. The complete label codebooks for triage departments, diagnosis categories, and treatment options are presented in Table S2–S4 of the Supplementary Material, respectively. These resources provide explicit definitions for every label, precise inclusion and exclusion criteria, and clearly articulated rules for resolving ambiguities among overlapping services.

**Table 3:** Data source of the IPDS benchmark

Input	Source	Detailed file
language, marital status, race	MIMIC-IV-Hosp	admissions.csv
gender	MIMIC-IV-Hosp	patients.csv
icd_code, icd_version, long_title, disease	MIMIC-IV-Hosp	d_icd_diagnoses.csv
curr_service	MIMIC-IV-Hosp	services.csv
medical history	MIMIC-IV-Note	discharge.csv
radiological report	MIMIC-IV-Note	radiology.csv
department	MIMIC-IV-ICU	icustays.csv

**Table 4:** Triage options in the IPDS benchmark

Abbreviation	Details
CVICU	Cardiac Vascular Intensive Care Unit
CCU	Coronary Care Unit
MICU	Medical Intensive Care Unit
MSICU	Medical and Surgical Intensive Care Unit
Neuro Intermediate	Neuro Intermediate
Neuro Stepdown	Neuro Stepdown
Neuro SICU	Neuro Surgical Intensive Care Unit
SICU	Surgical Intensive Care Unit
TSICU	Trauma Surgical Intensive Care Unit

**Table 5:** Diagnosis options in the IPDS benchmark

Abbreviation	Details
D1	Symptoms, signs, and abnormal clinical and laboratory findings, not elsewhere classified
D2	Symptoms, signs, and abnormal clinical and laboratory findings
D3	Pregnancy, childbirth and the puerperium
D4	Neoplasms
D5	Mental and behavioral disorders
D6	Injury, poisoning, and certain other consequences of external causes
D7	Endocrine, nutritional and metabolic diseases
D8	Diseases of the skin and subcutaneous tissue
D9	Diseases of the respiratory system
D10	Diseases of the nervous system and sense organs
D11	Diseases of the musculoskeletal system and connective tissue
D12	Diseases of the genitourinary system
D13	Diseases of the digestive system
D14	Diseases of the circulatory system
D15	Diseases of the blood and blood-forming organs and certain disorders involving the immune mechanism
D16	Congenital malformations, deformations and chromosomal abnormalities
D17	Certain infectious and parasitic diseases

**Table 6:** Treatment options in the IPDS benchmark

Index	Detailed text
T1	Vascular surgery, mainly refers to surgery related to the circulatory system
T2	Thoracic surgery, mainly refers to chest surgery between the abdomen and the neck
T3	Trauma surgical treatment, physical injury or damage caused by external physical factors
T4	General surgical treatment, mainly refers to types of surgery that cannot be classified by speciality
T5	Plastic treatment, mainly for the repair or reconstruction of the human body
T6	Orthopedic surgical treatment, mainly involving the musculoskeletal system
T7	Orthopedic treatment, mainly involving the musculoskeletal system
T8	Obstetrics, maternal classification and refusal
T9	Neurosurgery treatment, surgical treatment related to the brain
T10	Neurology treatment, non-surgical treatment related to the brain
T11	General medical treatment
T12	Gynecological treatment, female reproductive system and breasts, etc.
T13	Urogenital treatment, urinary system and reproductive system
T14	Otolaryngology treatment mainly for the ear, nose and throat-related areas
T15	Cardiovascular surgery treatment, surgical treatment of cardiovascular diseases
T16	Cardiovascular medicine treatment, conservative treatment of cardiovascular diseases

Using unique subject identifiers, we performed multi-table search and integration operations to construct the IPDS benchmark, comprising 51,274 patient cases. Each case incorporates curated demographic information, radiological reports, and medical history. The task labels in our IPDS benchmark were inherited from the clinical records of the public MIMIC-IV 2.2 database, which provides categories based on clinical demands and real-world usage. On this basis, our goal is to establish classification tasks that not only reflect real clinical workflows but also optimize medical decision-making efficiency and resource allocation. While a few categories might appear broad or overlapping, their primary purpose is to simplify complex medical tasks, particularly in high-pressure settings such as emergency departments or intensive care units, and to support rapid decision-making^63–69^.

In the triage task, we adopted the label of the assigned clinic (i.e., the first_careunit column in icustays.csv) to which the patient was first admitted as the triage label. As such, the distinctions among *Medical Intensive Care Unit* (MICU), *Surgical Intensive Care Unit* (SICU), and Medical and *Surgical Intensive Care Unit* (MSICU) were directly based on the actual departmental categories in the MIMIC-IV database, reflecting the management needs of different specialized critical care patients. Typically, MICU handles cases requiring advanced medical interventions (e.g., sepsis or respiratory failure), SICU focuses on post-surgical monitoring and care, while MSICU manages complex cases requiring both medical and surgical interventions. Although these categories may have some degree of overlap, they represent the actual resource allocation in medical institutions. Such classifications help optimize triage workflows, improve resource utilization efficiency, and ensure that medical teams can respond quickly to high-priority and high-demand cases.

In the diagnosis task, we collected the ICD code records (i.e., the icd_code and icd_version columns in diagnoses_icd.csv), and sorted the diagnosis labels based on the taxonomy of the World Health Organization^70–72^. This taxonomy balances clinical practicality, scientific logic, and management efficiency. In this way, the distinction between *D1: Symptoms, signs, and abnormal clinical and laboratory findings, not elsewhere classified*, and *D2: Symptoms, signs, and abnormal clinical and laboratory findings* also originated from the label definitions in the MIMIC-IV data, aiming to capture common uncertainties in clinical diagnostics. More specifically, D1 is used to record symptoms or findings that are not yet definitively diagnosed, serving as a transitional classification during the diagnostic process, while D2 applies to symptoms and findings that are well-documented but not yet categorized under a specific disease. This classification provides clinicians with a structured diagnostic tool to address clinical uncertainties systematically, thereby improving diagnostic efficiency.

In the treatment task, we adopted the label of the treatment (i.e., the curr_service column in services.csv) to which the patient is taken after the diagnosis. As such, *T4: General surgical treatment* describes cases requiring surgical interventions (e.g., appendectomy or tumor resection), while *T11: General medical treatment* covers non-surgical interventions such as pharmacological therapy or supportive care. While some terms, such as general treatment, may seem broad, they provide a high-level perspective that helps clinical teams quickly identify patient intervention needs and coordinate resource allocation across departments. This classification is particularly important in emergency and ICU settings, where rapid decision-making is critical, and it can significantly enhance medical efficiency and resource utilization.

The data sanitization process was rigorous and multi-faceted, where the IPDS used random ciphers to replace patient identifiers and applied stringent rules to structured columns. Text fields were filtered using manually curated allow and block lists, and the IPDS implemented context-specific regular expressions. A free-text benchmark sanitization algorithm was applied, followed by a manual review to ensure the complete removal of personally identifiable information. This thorough process ensured patient privacy while maintaining the clinical information and integrity of the data. The dataset was distributed as comma-separated value files, with each row representing a unique case identified by a unique subject_id and hadm_id. Access to the IPDS required registration on PhysioNet, identity verification, completion of human participant training, and signing of a data use agreement. These measures ensured the ethical use of the data while maintaining its research value. To facilitate research and collaboration, we established an open-source MIMIC-IV Code Repository, serving as a platform for shared discussion and analysis of all versions of MIMIC, including the IPDS benchmark.

### Dataset construction and preprocessing pipeline

The specific preprocessing scripts for constructing the dataset from the MIMIC-IV database were implemented using Python v3.10 and the Pandas v2.1.3 library. These scripts performed multi-table searches and integration based on unique subject identifiers (subject_id and hadm_id) to consolidate records from MIMIC-IV, including admissions.csv, patients.csv, d_icd_diagnoses.csv, services.csv, discharge.csv, radiology.csv, and icustays.csv. To ensure methodological rigor, the data preprocessing and screening logic were executed through a systematic, multi-step pipeline. First, during the case selection phase, the scripts extracted only the first hospital visit for each patient to guarantee patient-level sample independence and avoid overlap or redundancy from subsequent visits. Following this, the pipeline performed standardized label mapping and categorization by implementing the structured label codebooks detailed in the supplementary material. Specifically, the code utilized (Table S1) to precisely map raw ICD-9 and ICD-10 codes into 17 standardized disease categories (D1–D17) based on explicit block ranges. The scripts further applied strictly defined inclusion and exclusion criteria to categorize patients into specific triage departments (Table S2), diagnosis categories (Table S3), and standardized treatment options (Table S4), thereby effectively resolving ambiguities among overlapping clinical services. Subsequently, data consolidation and screening were conducted to identify and handle missing values, specifically filtering out samples with null radiological reports. This step also involved comprehensive data screening to remove irrelevant distractors, such as medical history information completely unrelated to the on-site radiological findings. Finally, the pipeline enforced a rigorous benchmark sanitization process. This concluding stage entailed replacing original identifiers with random ciphers, applying manually curated allow and block lists for text fields, implementing context-specific regular expressions, and utilizing a free-text benchmark sanitization algorithm to prevent data leakage.

### The MAP framework

The Multi-Agent Inpatient Pathways (MAP) framework, illustrated in Fig. 2, implemented a Triage-Diagnosis-Treatment (TDT) clinical pathway through collaboration among three clinician agents and a chief agent. In the MAP framework, each agent was empowered by a specialized LLM augmented with domain-specific medical knowledge (e.g., ICD-10 codes, NICE guidelines), and was capable of processing and understanding complex medical scenarios. We organized the communication among agents using a structured protocol with three fields, including the context, thinking, and answer. The context field contained a brief summary of the current task and the conversation history among agents. The thinking field was composed of the reasoning of the agent and provided transparency to help other agents understand better. The answer field was the decision of the agent in the format of a concise description for standardized medical terminology with ICD-10 codes. The structured protocol makes the interaction of agents well-informed and efficient, which is traceable and interpretable in clinical deployment.

Along the inpatient pathway, the first Triage Agent prioritized patient urgency by comprehensively analyzing radiology reports (e.g., Modified Early Warning System) and medical history (e.g., Hypertension, Hyperlipidemia, Atrial Fibrillation/flutter), and assigned patients to one of nine departments (e.g., Coronary Care Unit). Then, the Diagnosis Agent, deployed within the specific department, took the output of the first Triage Agent and the demographic information, radiological reports, medical history, routine examination, and preliminary evaluation results as input and employed chain-of-thought reasoning to generate accurate diagnoses. Afterwards, the Treatment Agent continued to determine the appropriate treatment plan for the patient based on the analysis provided by the preceding two clinical agents and the patient’s information, and completed the entire inpatient pathway process. These three clinician agents were enhanced with our tailored modules, including the record review module, the trainable retrieval-enhanced generation module, and the expert guidance module. In particular, the Chief Agent, involved in the expert guidance module during the training, took the patient’s condition and was responsible for overseeing these clinician agents, judging whether their predictions of the triage, diagnosis and treatment were convincing or not. The Chief Agent would point out the mistakes and improper reasoning effectively, and provide structured guidance to remind the clinician agents to reconsider and learn from such supervision. Notably, the record review, trainable retrieval-enhanced generation, and expert guidance modules, are designed to be fundamentally label-agnostic, which ensures that the pipeline can be effectively adapted to other clinical taxonomies, such as the new version of ICD, or applied to different hospital-specific classification systems.

### The record review module

The record review module was designed to select the beneficial input data (e.g., the medical history records and radiological reports), aiming to address cases where the medical history records contradict the radiological reports. This procedure ensures that only relevant and meaningful data are considered in the diagnostic process. Specifically, we used ClinicalBERT^73^ to embed the medical entities and the overall report into a correlation coefficient matrix, where each element represents the degree of correlation between a specific medical fact in the medical history and the features in the radiology report. Then, we applied the cosine similarity function to calculate the importance score of each entity. The key entities with high scores were used as input for correlation analysis. For example, an inpatient case with a high correlation score is calculated when the radiology report emphasizes lung abnormalities and the medical history mentions *pulmonary nodules*. We set a threshold for the correlation coefficient (empirically set as 0.1), and considered medical history with a correlation coefficient lower than the correlation threshold to be irrelevant to the radiology report, thus only considering reliable information from the radiology report for the clinical decision. Therefore, the record review module ensures that irrelevant or noisy medical history data do not interfere with diagnostic reasoning with the on-site radiological report, improving the quality of input data for diagnosis and reducing the risk of errors caused by unrelated information.

### The trainable retrieval-enhanced generation module

The trainable retrieval-enhanced generation module dynamically adapted to domain-specific datasets and task-specific requirements by integrating a fine-tuned retriever and the LLM within an end-to-end framework. For each patient case, the retriever processed diverse input data, including demographic information, radiological reports, and medical history, to retrieve highly relevant documents. The knowledge base was constructed from a comprehensive collection of real-world clinical cases and authoritative medical guidelines, such as those provided by the National Institute for Health and Care Excellence (NICE)^74^. Using the vector store architecture of LlamaIndex^75^, all documents in the knowledge base were embedded into dense vector representations through a pre-trained language model, such as ClinicalBERT^73^, fine-tuned on clinical datasets to capture nuanced semantic relationships specific to the medical domain. The retriever leveraged these embeddings to perform semantic search, identifying the top 10 most relevant documents for each patient case based on cosine similarity. To ensure that the retriever aligns with the task-specific requirements, the module employed a joint optimization approach where gradients from the LLM’s loss (e.g., cross-entropy on diagnostic reasoning outputs) were propagated back to fine-tune the retriever’s parameters, enabling dynamic adaptation to the input data and task objectives. To further enhance diagnostic reasoning capabilities, a structured Chain-of-Thought (CoT)^76^ reasoning tool was incorporated, which integrated diagnostic reasoning steps, the derived diagnosis, and supporting evidence into the input data. This structured reasoning not only ensures that the training data includes highly relevant similar cases and medical guidelines but also improves interpretability and robustness by providing transparent and clinically sound diagnostic conclusions.

### The expert guidance module

The expert guidance module was designed to provide guidance to facilitate the reasonable diagnostic process performed by the diagnosis agent. Specifically, a chief agent was designed to systematically evaluate the diagnosis agent’s diagnostic conclusions against established medical standards and guidelines of the knowledge base, i.e., it verified whether the proposed diagnosis was supported by sufficient evidence, aligned with clinical guidelines, and adhered to standard diagnostic procedures. When inconsistencies in the diagnostic reasoning were detected, the chief agent pinpointed the specific issues. For example, if the diagnosis agent overlooked the crucial lung nodule feature in the radiological report, the chief agent may generate guidance: *The lung nodule feature mentioned in the radiological report seems relevant to this case but was not considered in the diagnostic reasoning*. In summary, the expert guidance module introduced guidance and reflection on the reasoning process through the knowledge base, enhancing the diagnostic reasoning capability of the model.

### Implementation details

All experiments were performed on a Linux platform with eight NVIDIA A800 GPUs. We selected LLaMA3-8B as the base model for four specialized agents in our MAP framework. Three clinician agents equipped with three tailored modules were optimized with 3 epochs with a learning rate of 2 × 10^−5^, and the chief agent was equipped with a standard retrieval-augmented generation with real patient cases from clinical practice alongside authoritative medical guidelines to provide high-quality guidance. During the training, we froze the LLM parameters while fine-tuning the learnable parameters in the trainable retrieval-enhanced generation (REG) module to avoid overfitting. During the inference, three clinician agents in the MAP framework are evaluated with the record review module and the trainable retrieval-enhanced generation module, since the expert guidance module is designed to enhance the training process.

To align with clinical workflows and ensure a fair comparison with clinicians, we implemented a multi-label classification design for the diagnosis and treatment tasks, allowing up to top-3 predictions. This design reflects clinical practices where multiple potential diagnoses or treatments are often considered, and ensures reproducible and meaningful comparisons between the MAP framework and clinicians. Additionally, clinicians were provided with detailed training protocols, including task definitions, label descriptions, and their alignment with clinical practice, to minimize potential biases and ensure consistency in evaluations.

### Evaluation metrics

To comprehensively evaluate the performance of LLMs in the inpatient setting, we employed a series of evaluation metrics, including accuracy (Acc), macro F1-score (F1)^77^, Area Under the receiver operating characteristic Curve (AUC)^78^, macro precision (PRE), sensitivity (SEN)^79^, specificity (SPE)^80^, Cohen’s Kappa (CK)^81^, and Matthews Correlation Coefficient (MCC)^82^. Moreover, to meet the needs of clinical diagnosis, we utilized the Disease-Specific Misdiagnosis Rate (DSMR), which quantified the proportion of misdiagnosed cases (false positives) relative to the total number of actual cases for a specific disease. It could be mathematically expressed as DSMR = *n*_*misd*/*n*_*total* × 100%, where *n*_*misd* denotes the number of misdiagnoses with both false positives and false negatives, *n*_*total* denotes the number of diagnoses. We also applied the two-way random effects Intraclass Correlation Coefficient ICC(2, *k*)^83^ to analyze the rating reliability since it is well-suited to that case multiple raters independently evaluate the same subjects. It could be mathematically expressed as (MSR − MSE)/(MSR + (1/*n*) × (*k* ⋅ MSC − MSE)), where MSR, MSE, MSC, *n*, and *k* denote the Mean Square for Rows, Mean Square Error, Mean Square for Columns, the number of subjects, and the number of raters, respectively. The ICC reference table is as follows: ICC < 0.40: Poor reliability; 0.40≤ICC < 0.60: Fair reliability; 0.60≤ICC < 0.75: Good reliability; ICC≥0.75: Excellent reliability.

### Ablation study and qualitative case study

**Fig. 9:**
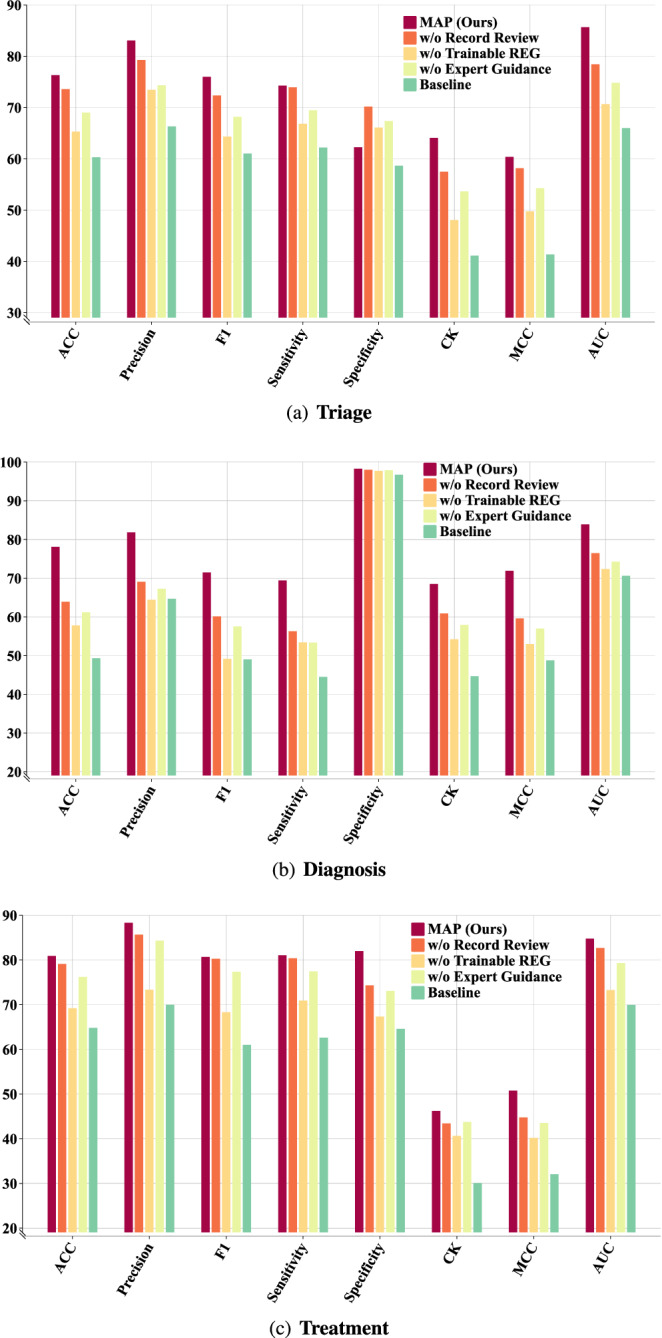
Comprehensive ablation studies of the record review, trainable REG, and expert guidance modules on inpatient pathway tasks with eight evaluation metrics. **a** Triage, **b** diagnosis, and **c** treatment.

**Fig. 10:**
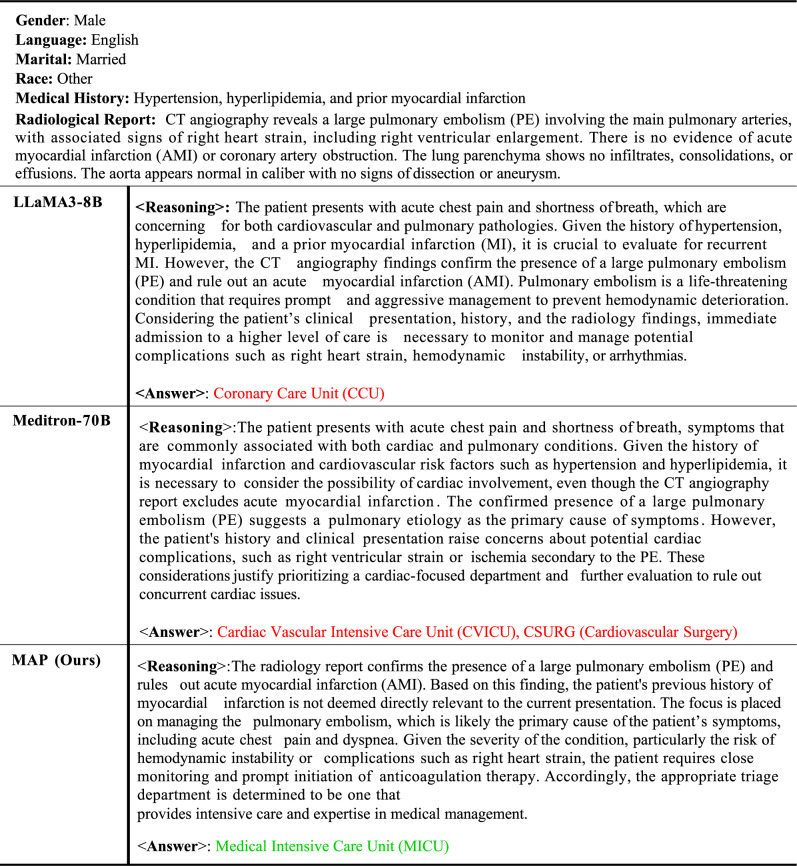
The comparison shows the triage and its reasoning of LLaMA3-8B, Meditron-70B, and our MAP. For better visualization, we highlight the incorrect parts in red and the correct parts in green.

**Fig. 11:**
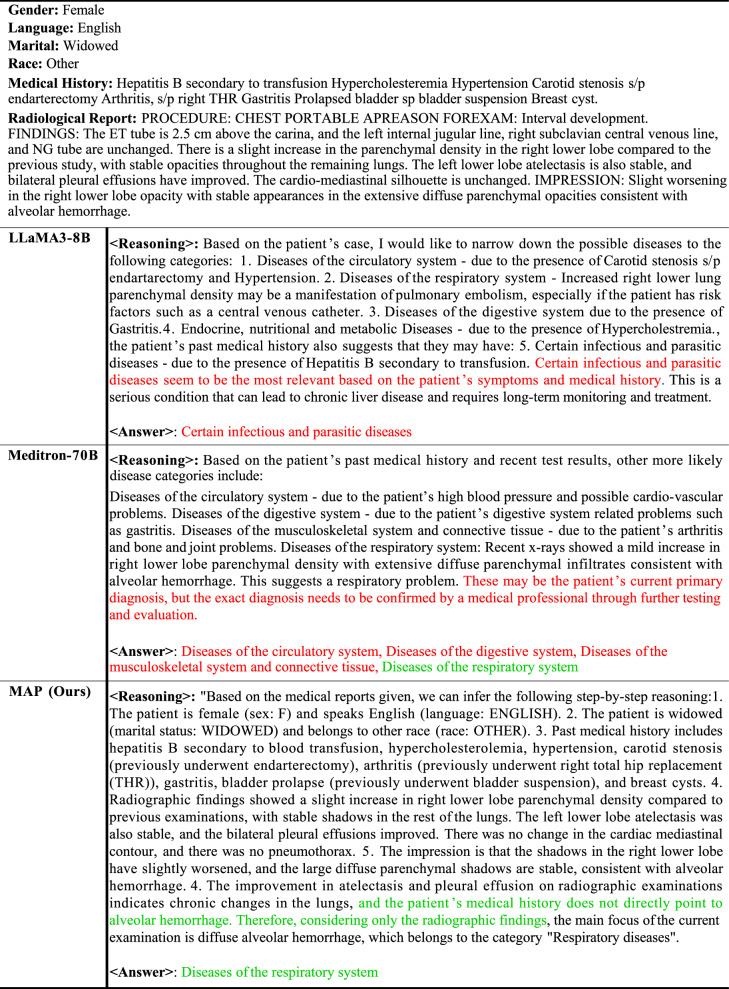
The comparison shows the diagnosis and its reasoning of LLaMA3-8B, Meditron-70B, and our MAP. For better visualization, we highlight the incorrect parts in red and the correct parts in green.

**Fig. 12:**
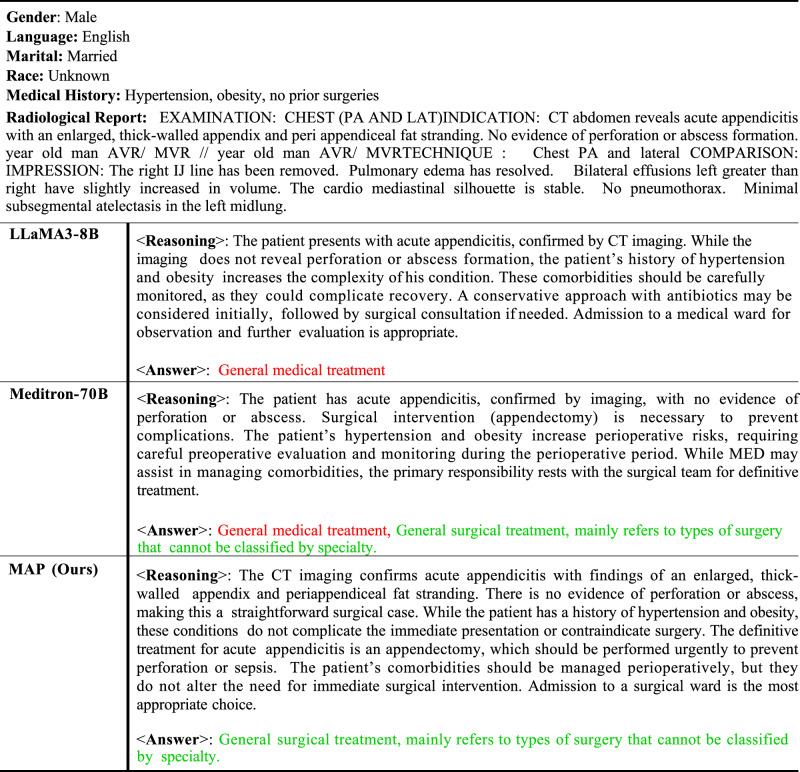
The comparison shows the treatment and its reasoning of LLaMA3-8B, Meditron-70B, and our MAP. For better visualization, we highlight the incorrect parts in red and the correct parts in green.

Comprehensive ablation studies in Fig. 9 were conducted to evaluate the module effectiveness across inpatient pathway tasks. We compared the performance in eight evaluation metrics and confirmed that the proposed MAP framework reveals consistent improvements over ablative baselines in most scenarios, as shown in Figs. 10–12. For instance, in Fig. 11, the baseline LLaMA3-8B model tended to overemphasize medical history (e.g., infectious diseases) and overlooked critical radiographic findings (e.g., stable shadows in the lungs). In contrast, our MAP framework explicitly reasoned that the medical history has no direct correlation with current alveolar hemorrhage, demonstrating a structured and professional diagnostic approach. This structured reasoning process enabled the model to achieve more accurate diagnoses, particularly for respiratory diseases. The results highlight its ability to align reasoning patterns with professional clinical practice, effectively integrating diverse information sources into a coherent diagnostic framework (Table 6). These systematic comparisons identified key imaging abnormalities and appropriately weighted different sources of information.

## Supplementary information

Supplementary Information

## Data Availability

The dataset is available to all researchers who create an account on https://physionet.org/ and follow the steps to gain access to the MIMIC-IV database (https://physionet.org/content/mimiciv/3.0/). Access is given after being a credentialed user and completing the “CITI Data or Specimens Only Research” training course. The data use agreement of PhysioNet for the project must also be signed. The generated dataset can also be directly downloaded from PhysioNet.

## Code availability

The evaluation framework used for this study can be found at GitHub (https://github.com/franciszchen/MAP/). The analysis framework to evaluate all results, generate all plots, and perform all statistical analyses can be found at Google Drive (https://t.ly/N0JVT). All code uses Python v3.10, PyTorch v2.1.2, Transformers v4.42.4, Spacy v3.7.5, LangChain v0.0.339, Llama-index-core v0.10.15, vllm v0.4.0+cu118, NLTK v3.8.1 and OpenAI v1.6.1. The code to create the dataset uses Python v3.10 and Pandas v2.1.3.
