# Supplementary material for: MAP: evaluation and multi-agent enhancement of large language models for inpatient pathways
Source: Npj Health Syst. 2026 Jun 1;3:37. doi: 10.1038/s44401-026-00085-0 (PMC13336800; doi:10.1038/s44401-026-00085-0)
Supplement: Supplementary file 1 — Supplementary Information [file 44401_2026_85_MOESM1_ESM.pdf]

Supplementary for MAP: Evaluation and Multi-Agent Enhancement of Large Language Models for Inpatient Pathways

Table S1: **Diagnosis mapping codebook.** Detailed mapping between the standardized disease categories (D1–D17) and the corresponding ICD-10 and ICD-9 code ranges.

Label	ICD Version	Block Range	Standardized Detail
D1	ICD-10	R00–R99	Symptoms, signs and abnormal clinical and laboratory findings, not elsewhere classified
D2	ICD-9	780–799	Symptoms, signs and Ill-defined conditions
D3	ICD-10	O00–O99	Pregnancy, childbirth and the puerperium
	ICD-9	630–679	Complications of pregnancy, childbirth, and the puerperium
D4	ICD-10	C00–D48	Neoplasms
	ICD-9	140–239	
D5	ICD-10	F00–F99	Mental and behavioural disorders
	ICD-9	290–319	Mental disorders
D6	ICD-10	S00–T98	Injury, poisoning and certain other consequences of external causes
	ICD-9	800–999	Injury and poisoning
D7	ICD-10	E00–E90	Endocrine, nutritional and metabolic diseases
	ICD-9	240–279	Endocrine, nutritional and metabolic diseases, and immunity disorders
D8	ICD-10	L00–L99	Diseases of the skin and subcutaneous tissue
	ICD-9	680–709	
D9	ICD-10	J00–J99	Diseases of the respiratory system
	ICD-9	460–519	
D10	ICD-10	G00–G99	Diseases of the nervous system
	ICD-9	320–389	Diseases of the nervous system and sense organs
D11	ICD-10	M00–M99	Diseases of the musculoskeletal system and connective tissue
	ICD-9	710–739	
D12	ICD-10	N00–N99	Diseases of the genitourinary system
	ICD-9	580–629	
D13	ICD-10	K00–K93	Diseases of the digestive system
	ICD-9	520–579	
D14	ICD-10	I00–I99	Diseases of the circulatory system
	ICD-9	390–459	
D15	ICD-10	D50–D89	Diseases of the blood and blood-forming organs and certain disorders involving the immune mechanism
	ICD-9	280–289	Diseases of the blood and blood-forming organs
D16	ICD-10	Q00–Q99	Congenital malformations, deformations and chromosomal abnormalities
	ICD-9	740–759	Congenital anomalies
D17	ICD-10	A00–B99	Certain infectious and parasitic diseases
	ICD-9	001–139	Infectious and parasitic diseases

Table S2: **Details of the triage label codebook.**

Label	Detail	Definition	Inclusion Criteria	Exclusion Criteria
CVICU	Cardiac Vascular Intensive Care Unit	Surgical & Complex Medical Heart/Vascular Care. Manages the most critical patients, often post-operative from major cardiac/vascular surgeries (e.g., open-heart, valve replacements) or with complex medical heart failure requiring advanced life support.	The detailed inclusion criteria is patients with critical cardiac or vascular illness immediately after major surgery or requiring mechanical circulatory support. For example, post-heart transplant, post-aortic dissection repair with complications, cardiogenic shock on multiple vasopressors plus mechanical support, acute prosthetic valve failure.	The detailed exclusion criteria is patients whose primary problem is not post-cardiac surgery or device-dependent cardiogenic shock. For example, stable post-cardiac surgery postoperative day ≥3 (→ cardiac stepdown), primary sepsis or acute respiratory distress syndrome (ARDS) without cardiac surgery (→ MICU).
CCU	Coronary Care Unit	Medical Coronary Artery Disease. Specializes in managing acute, non-surgical coronary events like myocardial infarctions (heart attacks), unstable angina, and serious arrhythmias, typically with intensive medication and monitoring.	The detailed inclusion criteria is acute coronary events and malignant arrhythmias without the need for cardiac surgery. For example, unstable angina with ongoing ischemia, post-cardiac arrest with primary coronary etiology.	The detailed exclusion criteria is any patient requiring or recovering from cardiac surgery or mechanical circulatory support. For example, any post-cardiac surgery patient (→ CVICU), primary non-coronary shock (→ MICU).
MICU	Medical Intensive Care Unit	Non-Surgical Medical Crises. Manages life-threatening medical conditions such as severe sepsis, respiratory failure requiring intubation, metabolic comas, and multi-organ dysfunction from medical (not surgical) causes.	The detailed inclusion criteria is life-threatening medical conditions without a surgical source. For example, septic shock from pneumonia, acute liver failure, myasthenic crisis, drug overdose requiring intubation.	The detailed exclusion criteria is any patient whose primary critical illness is post-operative or trauma-related. For example, isolated acute coronary syndrome (→ CCU), polytrauma (→ TSICU).
MSICU	Medical and Surgical Intensive Care Unit	Mixed Patient Population. A hybrid unit that cares for a broad range of critically ill patients, regardless of whether their primary issue is medical or surgical, serving as a general-purpose ICU.	The detailed inclusion criteria is any critically ill patient when the hospital lacks dedicated specialized ICUs. For example, post-laparotomy with sepsis, medical ARDS, minor trauma, septic shock from any source in a small hospital.	The detailed exclusion criteria is applied only in hospitals that possess specialized ICUs; highly specialized cases must be transferred out. For example, post-cardiac surgery (→ CVICU), major polytrauma (→ TSICU), post-craniotomy (→ Neuro SICU).
Neuro Intermediate	Neuro Intermediate	Stable Neurological Monitoring. A step-down unit for neurologically stable patients who no longer need intensive care but require more frequent neurological checks and nursing care than a general ward provides (e.g., stable stroke patients).	The detailed inclusion criteria is acute brain/spine injury or post-neurosurgery patients needing intensive neuromonitoring or intervention. For example, refractory status epilepticus, acute spinal cord injury with neurogenic shock.	The detailed exclusion criteria is neurologically stable patients or isolated medical critical illness without neuro-critical needs. For example, stable stroke >72 h (→ Neuro Stepdown), medical sepsis without neurological involvement (→ MICU).
Neuro Stepdown	Neuro Stepdown	Transitional Neurological Care. Functions as a direct transitional unit for patients recently discharged from the Neuro SICU or SICU. These patients are out of critical condition but still have a high risk of rapid neurological deterioration and need very close monitoring.	The detailed inclusion criteria is neurocritical ill patients recently stepped down from Neuro SICU but still requiring close monitoring. For example, Guillain-Barré weaning from ventilator.	The detailed exclusion criteria is patients still requiring full ICU-level neuro interventions or those already stable for transfer to intermediate/floor. For example, active intracranial pressure management or hourly neuro checks (→ Neuro SICU).
Neuro SICU	Neuro Surgical Intensive Care Unit	Post-Neurosurgical & Acute Brain/Spine Trauma. Dedicated to the immediate, critical care following brain or spinal surgery, and for managing severe traumatic brain or spinal cord injuries, including intracranial pressure monitoring.	The detailed inclusion criteria is neurologically stable patients requiring intermediate-level monitoring only. For example, ischemic stroke day 3–10 on telemetry, controlled seizures on oral medications, post-carotid endarterectomy observation.	The detailed exclusion criteria is any patient requiring intensive interventions or frequent neuro checks. For example, continuous vasoactive infusions, mechanical ventilation, q1h neuro checks, or unstable neurology (→ Neuro Stepdown).
SICU	Surgical Intensive Care Unit	General, Abdominal, and Transplant Surgery. Manages critically ill patients recovering from major non-cardiac, non-neurological surgeries, such as major abdominal, trauma, or transplant surgeries, focusing on surgical complication management.	The detailed inclusion criteria is critically ill patients recovering from major abdominal, transplant (non-heart), or vascular (non-cardiac) surgery. For example, post-Whipple or esophagectomy, liver/kidney/pancreas transplant, necrotizing pancreatitis, abdominal compartment syndrome.	The detailed exclusion criteria is cardiac surgery, neurosurgery, major trauma, and burns. For example, post-cardiac surgery (→ CVICU), post-craniotomy (→ Neuro SICU), polytrauma (→ TSICU).
TSICU	Trauma Surgical Intensive Care Unit	Multi-System Blunt/Penetrating Trauma. A specialized SICU for the initial resuscitation and comprehensive management of patients with severe, multi-system traumatic injuries (e.g., from car accidents, falls, gunshot wounds), often involving coordinated care between surgical specialties.	The detailed inclusion criteria is severe polytrauma or penetrating injury affecting multiple body systems. For example, damage-control laparotomy after trauma, traumatic hemoperitoneum + multiple fractures.	The detailed exclusion criteria is isolated single-system injury or non-traumatic critical illness. For example, isolated traumatic brain injury (→ Neuro SICU).

Table S3: **Details of the diagnosis label codebook.**

Label	Detail	Definition	Inclusion Criteria	Exclusion Criteria
D1	Symptoms, signs, and abnormal clinical and laboratory findings, not elsewhere classified	Symptoms, signs or clinical findings that do not have a definite diagnosis and are not more appropriately classified in another chapter	The detailed inclusion criteria is non-specific symptoms, abnormal findings, or transient signs without confirmed etiology. For example, fever of unknown origin, generalized weakness, abnormal liver function tests of undetermined cause, asymptomatic bacteriuria, incidental radiologic finding.	The detailed exclusion criteria is any symptom/sign that has a confirmed etiology or belongs to another specific chapter. For example, hemoptysis due to lung cancer (→ D4), jaundice due to gallstones (→ D13), seizure due to epilepsy (→ D10).
D2	Symptoms, signs, and abnormal clinical and laboratory findings	Reserved in some national modifications; usually merged into D1 or used for provisional coding when information is incomplete	The detailed inclusion criteria is provisional or incomplete diagnostic statements awaiting confirmation. For example, rule out sepsis, observe for head injury.	The detailed exclusion criteria is any case with final confirmed diagnosis. Almost always reclassified to specific chapter upon discharge.
D3	Pregnancy, childbirth and the puerperium	All conditions associated with pregnancy, delivery, and the postpartum period (usually up to 42 days post-delivery)	The detailed inclusion criteria is any maternal condition or complication directly related to pregnancy/delivery/puerperium. For example, gestational diabetes, pre-eclampsia, cesarean section, postpartum hemorrhage, puerperal sepsis, miscarriage, ectopic pregnancy.	The detailed exclusion criteria is fetal/newborn conditions and non-pregnancy-related diseases in pregnant women. For example, appendicitis in pregnant woman (→ D13), pre-existing hypertension (→ D14).
D4	Neoplasms	All benign, malignant, in-situ, and uncertain-behavior neoplasms, including secondary/metastatic sites	The detailed inclusion criteria is any new or existing tumor regardless of behavior. For example, lung adenocarcinoma, colon cancer with liver metastasis, meningioma, leukemia, lymphoma, carcinoma in situ of cervix, myelodysplastic syndrome.	The detailed exclusion criteria is functional endocrine tumors coded by symptoms and secondary malignancies coded by primary site. For example, hyperthyroidism due to toxic adenoma (→ D7), metastatic site is additional code, not primary.
D5	Mental and behavioral disorders	Psychiatric disorders, including substance use disorders and behavioral syndromes	The detailed inclusion criteria is primary psychiatric and substance-related conditions. For example, schizophrenia, major depressive disorder, bipolar disorder, alcohol dependence, opioid use disorder, dementia, anorexia nervosa.	The detailed exclusion criteria is neurologic diseases with psychiatric features and secondary mental symptoms. For example, depression due to hypothyroidism (→ D7).
D6	Injury, poisoning, and certain other consequences of external causes	Fractures, burns, trauma, poisoning, adverse effects, complications of surgery and medical care	The detailed inclusion criteria is conditions caused by external physical or chemical agents. For example, traumatic subdural hematoma, femoral fracture, thermal burn, opioid overdose, anaphylaxis to penicillin, postoperative wound infection.	The detailed exclusion criteria is internal diseases and late effects already in other chapters. For example, ischemic stroke (→ D14), osteoporosis with pathological fracture (→ D11), diabetic foot ulcer (→ D7).
D7	Endocrine, nutritional and metabolic diseases	Diabetes, thyroid disorders, obesity, malnutrition, metabolic syndrome, inborn errors of metabolism	The detailed inclusion criteria is disorders of glands, nutrition, and metabolism. For example, type 1/2 diabetes, hypothyroidism, Cushing syndrome, obesity, hyperlipidemia, phenylketonuria.	The detailed exclusion criteria is endocrine tumors and secondary endocrine dysfunction. For example, insulinoma (→ D4), hypercalcemia due to lung cancer (→ D4).
D8	Diseases of the skin and subcutaneous tissue	Dermatitis, psoriasis, infections of skin, pressure ulcers, cellulitis	The detailed inclusion criteria is primary diseases of skin and subcutaneous tissue. For example, atopic dermatitis, psoriasis, cellulitis, decubitus ulcer, pemphigus, urticaria.	The detailed exclusion criteria is skin manifestations of systemic disease. For example, diabetic dermopathy (→ D7).
D9	Diseases of the respiratory system	COPD, asthma, pneumonia, pulmonary embolism, lung fibrosis, pleural effusion	The detailed inclusion criteria is primary respiratory tract and lung parenchyma diseases. For example, community-acquired pneumonia, asthma attack, pulmonary fibrosis, bronchiectasis, pneumothorax.	The detailed exclusion criteria is cardiac, neoplastic, and trauma-related lung disease. For example, pulmonary edema due to heart failure (→D14), lung cancer (→D4), hemothorax (→D6).
D10	Diseases of the nervous system and sense organs	Epilepsy, stroke, migraine, Parkinson, glaucoma, otitis media, etc.	The detailed inclusion criteria is primary neurologic and special sensory (eye/ear) disorders. For example, ischemic stroke, epilepsy, Parkinson disease, multiple sclerosis, glaucoma, Ménière disease, retinal detachment.	The detailed exclusion criteria is trauma, tumor, and vascular surgery-related neurologic conditions. For example, traumatic brain injury (→ D6), brain tumor (→ D4), subarachnoid hemorrhage (→ D14).
D11	Diseases of the musculoskeletal system and connective tissue	Arthritis, osteoporosis, systemic lupus, spondylosis, gout	The detailed inclusion criteria is non-traumatic musculoskeletal and connective tissue diseases. For example, rheumatoid arthritis, osteoarthritis, systemic lupus erythematosus, osteoporosis, ankylosing spondylitis, gouty arthritis.	The detailed exclusion criteria is traumatic fractures and infections. For example, femoral neck fracture after fall (→ D6), septic arthritis (→ D17).
D12	Diseases of the genitourinary system	CKD, urinary tract infection, nephrolithiasis, BPH, gynecologic conditions in some classifications	The detailed inclusion criteria is primary renal, ureter, bladder, and male genital diseases. For example, chronic kidney disease, pyelonephritis, renal calculi, benign prostatic hyperplasia, urethral stricture.	The detailed exclusion criteria is gynecologic and pregnancy-related conditions. For example, pregnancy with UTI (→ D3).
D13	Diseases of the digestive system	Gastritis, cirrhosis, appendicitis, cholecystitis, pancreatitis	The detailed inclusion criteria is diseases of esophagus, stomach, intestines, liver, gallbladder, pancreas. For example, peptic ulcer, Crohn disease, cirrhosis, acute pancreatitis, diverticulitis, hemorrhoids.	The detailed exclusion criteria is abdominal wall and trauma conditions. For example, mesenteric ischemia due to atrial fibrillation (→ D14).
D14	Diseases of the circulatory system	Ischemic heart disease, heart failure, hypertension, stroke (in some systems), venous thromboembolism	The detailed inclusion criteria is primary cardiac and vascular diseases. For example, acute myocardial infarction, congestive heart failure, atrial fibrillation, essential hypertension, deep vein thrombosis, aortic aneurysm.	The detailed exclusion criteria is secondary hypertension and vascular tumors. For example, traumatic aortic injury (→ D6).
D15	Diseases of the blood and blood-forming organs and certain disorders involving the immune mechanism	Anemia, hemophilia, agranulocytosis, immunodeficiency states	The detailed inclusion criteria is primary hematologic and immunologic disorders. For example, iron deficiency anemia, sickle cell disease, hemophilia, primary immunodeficiency, lymphadenopathy due to lymphoma is additional.	The detailed exclusion criteria is secondary blood abnormalities. For example, anemia of chronic kidney disease (→ D12), thrombocytopenia due to sepsis (→ D17).
D16	Congenital malformations, deformations and chromosomal abnormalities	Congenital heart defects, Down syndrome, cleft palate, spina bifida	The detailed inclusion criteria is structural or chromosomal anomalies present from birth. For example, ventricular septal defect, Down syndrome, congenital hydrocephalus, cleft lip/palate, Turner syndrome.	The detailed exclusion criteria is acquired deformities. For example, post-traumatic limb deformity (→ D6), scoliosis due to cerebral palsy (→ D10).
D17	Certain infectious and parasitic diseases	Bacterial, viral, fungal, parasitic infections; sepsis	The detailed inclusion criteria is proven or presumed infectious diseases. For example, tuberculosis, COVID-19, malaria, sepsis, bacterial meningitis, HIV (sometimes in D15), urinary tract infection, cellulitis.	The detailed exclusion criteria is carrier states and non-infectious inflammation. For example, asymptomatic, non-infectious uveitis (→ D10).

Table S4: **Details of the treatment label codebook.**

Label	Detail	Explanation	Inclusion Criteria	Exclusion Criteria
T1	Vascular surgery, mainly refers to surgery related to the circulatory system	Vascular surgery encompasses operative interventions targeting pathologies of the circulatory system, including arteries, veins, and lymphatic vessels.	The detailed inclusion criteria is surgical treatment of arteries/veins/lymphatics outside the heart and intracranial vessels. For example, carotid endarterectomy, open/endovascular Abdominal Aortic Aneurysm (AAA) repair, peripheral bypass, varicose vein surgery, mesenteric revascularization.	The detailed exclusion criteria is cardiac surgery, intracranial vascular surgery, and non-surgical vascular disease. For example, cerebral aneurysm clipping/coiling (→ T9).
T2	Thoracic surgery, mainly refers to chest surgery between the abdomen and the neck	Thoracic surgery involves surgical procedures addressing conditions within the thoracic cavity, situated between the abdominal and cervical regions.	The detailed inclusion criteria is surgery inside the chest cavity excluding the heart. For example, lobectomy/pneumonectomy, esophagectomy, mediastinal tumor resection, chest wall resection, lung volume reduction surgery.	The detailed exclusion criteria is cardiac surgery, cardiac-containing procedures, and abdominal procedures. For example, diaphragm surgery via abdomen (→ T4).
T3	Trauma surgical treatment, physical injury or damage caused by external physical factors	Trauma surgery focuses on the management of physical injuries resulting from external mechanical or environmental forces.	The detailed inclusion criteria is operative management of acute injuries caused by blunt or penetrating trauma. For example, splenectomy for traumatic rupture, damage-control laparotomy, penetrating neck exploration, traumatic amputation revision.	The detailed exclusion criteria is elective surgery and non-traumatic acute surgical disease. For example, elective cholecystectomy (→ T4), perforated peptic ulcer (→ T4), acute appendicitis without trauma history (→ T4).
T4	General surgical treatment, mainly refers to types of surgery that cannot be classified by specialty	General surgery comprises a broad range of surgical procedures not confined to organ-specific or system-specific specialties.	The detailed inclusion criteria is abdominal, breast, endocrine, and soft-tissue surgery not belonging to other subspecialties. For example, cholecystectomy, hernia repair, mastectomy, thyroidectomy, appendectomy, colectomy for cancer or diverticulitis, perforated viscus.	The detailed exclusion criteria is surgery belonging to defined subspecialties. For example, thoracic (→ T2), neurosurgery (→ T9), urology (→ T13), gynecology (→ T12), plastic reconstruction (→ T5).
T5	Plastic treatment, mainly for the repair or reconstruction of the human body	Plastic surgery is dedicated to the restoration, reconstruction, or alteration of human bodily structures for functional or aesthetic purposes.	The detailed inclusion criteria is reconstruction, cosmetic surgery, and complex wound management. For example, free flap reconstruction, burn reconstruction, breast reconstruction, cleft lip/palate repair, pressure sore coverage, abdominoplasty, rhinoplasty.	The detailed exclusion criteria is primary trauma resuscitation and oncologic resection without reconstruction. For example, initial trauma exploration (→ T3).
T6	Orthopedic surgical treatment, mainly involving the musculoskeletal system	Orthopedic surgical treatment involves operative management of disorders affecting the musculoskeletal system, including bones, joints, and connective tissues.	The detailed inclusion criteria is surgical treatment of bones, joints, ligaments, tendons, and spine (non-neurosurgical). For example, total joint arthroplasty, fracture fixation, spinal fusion for degenerative disease, otator cuff repair.	The detailed exclusion criteria is neurosurgical spine, trauma resuscitation, and non-surgical management. For example, intradural spinal tumor (→ T9), polytrauma initial management (→ T3), conservative fracture care (→ T7).
T7	Orthopedic treatment, mainly involving the musculoskeletal system	Orthopedic treatment encompasses both surgical and non-surgical approaches to address musculoskeletal system impairments.	The detailed inclusion criteria is conservative and minor procedural management of musculoskeletal conditions. For example, casting/splinting, joint aspiration/injection, osteoporosis management, physiotherapy prescription, bracing for scoliosis.	The detailed exclusion criteria is major orthopedic surgery and other specialty surgery. For example, joint replacement or spinal fusion (→ T6), hand surgery belonging to plastic (→ T5).
T8	Obstetrics, maternal classification and refusal	Obstetrics is the medical specialty concerned with pregnancy, childbirth, and the postpartum period, including maternal health categorization and treatment decisions.	The detailed inclusion criteria is care related to pregnancy and delivery. For example, cesarean section, normal vaginal delivery, preeclampsia/eclampsia management, postpartum hemorrhage, fetal monitoring.	The detailed exclusion criteria is gynecologic disease unrelated to pregnancy. For example, ovarian cancer, endometriosis surgery for fibroids (→ T12), infertility treatment without current pregnancy.
T9	Neurosurgery treatment, surgical treatment related to the brain	Neurosurgery involves surgical interventions for pathologies of the brain, spinal cord, and peripheral nerves.	The detailed inclusion criteria is surgical treatment of central and peripheral nervous system. For example, craniotomy for tumor/aneurysm/hematoma, spinal decompression/fusion for tumor or cord compression, peripheral nerve repair.	The detailed exclusion criteria is non-surgical neurology and extracranial vascular surgery. For example, stroke thrombolysis, multiple sclerosis (→ T10), carotid endarterectomy (→ T1).
T10	Neurology treatment, non-surgical treatment related to the brain	Neurology focuses on the diagnosis and non-surgical management of disorders affecting the nervous system.	The detailed inclusion criteria is medical management of neurological diseases. For example, stroke thrombolysis/secondary prevention, epilepsy medication adjustment, Parkinson disease treatment, migraine management, myasthenia gravis immunosuppression.	The detailed exclusion criteria is any neurosurgical procedure. For example, brain tumor resection, clipped aneurysm, spinal cord tumor (→ T9).
T11	General medical treatment	General medical treatment refers to broad, non-surgical clinical management of common adult diseases and health conditions.	The detailed inclusion criteria is internal medicine conditions not belonging to subspecialties. For example, community-acquired pneumonia, cellulitis, hypertension crisis, diabetes management, electrolyte disorders.	The detailed exclusion criteria is organ-specific subspecialty diseases. For example, acute MI (→ T16), new-onset atrial fibrillation with RVR (→ T16).
T12	Gynecological treatment, female reproductive system and breasts, etc.	Gynecological treatment addresses medical conditions of the female reproductive system and associated structures such as the breasts.	The detailed inclusion criteria is non-obstetric female reproductive and breast diseases. For example, hysterectomy for fibroids, ovarian cystectomy, endometrial ablation, mastectomy for breast cancer, pelvic organ prolapse repair.	The detailed exclusion criteria is pregnancy-related conditions and non-gynecologic breast reconstruction. For example, cesarean section (→ T8), post-mastectomy flap reconstruction (→ T5).
T13	Urogenital treatment, urinary system and reproductive system	Urogenital treatment involves medical care for disorders of the urinary tract and the reproductive organs.	The detailed inclusion criteria is urology and andrology diseases (both surgical and medical). For example, nephrectomy, prostatectomy for cancer, kidney stone lithotripsy, vasectomy, erectile dysfunction treatment.	The detailed exclusion criteria is gynecologic and pediatric urology belonging to other specialties. For example, ovarian or uterine pathology (→ T12), pediatric circumcisions or undescended testis in children (pediatric urology).
T14	Otolaryngology treatment mainly for the ear, nose and throat-related areas	Otolaryngology encompasses the diagnosis and treatment of diseases related to the ear, nose, throat, and adjacent head and neck structures.	The detailed inclusion criteria is ENT and head & neck diseases. For example, tonsillectomy, septoplasty, tympanoplasty, parotidectomy, laryngectomy, neck dissection for cancer, sinus surgery.	The detailed exclusion criteria is intracranial, ophthalmic, and cervical vascular procedures. For example, acoustic neuroma (→ T9), carotid endarterectomy (→ T1).
T15	Cardiovascular surgery treatment, surgical treatment of cardiovascular diseases	Cardiovascular surgery entails operative correction of structural or functional abnormalities of the heart and great vessels.	The detailed inclusion criteria is surgery on the heart and intrathoracic great vessels. For example, valve repair/replacement, heart transplant, ascending aorta replacement, Bentall procedure, minimally invasive mitral surgery.	The detailed exclusion criteria is coronary disease without surgery and peripheral vascular surgery. For example, Coronary Artery Bypass Grafting (CABG) (→ T15), medical MI management (→ T16), peripheral artery bypass (→ T1).
T16	Cardiovascular medicine treatment, conservative treatment of cardiovascular diseases	Cardiovascular medicine involves non-invasive and pharmacologic management of diseases affecting the heart and vascular system.	The detailed inclusion criteria is non-surgical management of cardiac and vascular diseases. For example, acute coronary syndrome medical therapy, heart failure titration, atrial fibrillation rate/rhythm control, hypertension, pulmonary embolism anticoagulation.	The detailed exclusion criteria is any cardiac or great-vessel surgery. For example, CABG (→ T15).
